# Consensus Guidelines for Intermittent Auscultation in United States Community Birth Settings

**DOI:** 10.1111/birt.70002

**Published:** 2025-07-04

**Authors:** Silke Akerson, Sarah Bradbury, Rosanna Davis, Wendy Gordon, Amy Romano, Holly Scholles

**Affiliations:** ^1^ Oregon Perinatal Collaborative Oregon USA; ^2^ Broadlawns Medical Center Des Moines Iowa USA; ^3^ California Association of Licensed Midwives San Leandro California USA; ^4^ Bastyr University Kenmore Washington USA; ^5^ Primary Maternity Care Milford Connecticut USA; ^6^ Birthingway College Beaverton Oregon USA

**Keywords:** community birth, fetal assessment, intermittent auscultation, midwifery

## Abstract

**Background:**

Intermittent auscultation is the gold standard for fetal assessment in uncomplicated pregnancies and labors and is used universally in the community birth setting. Great variation exists in intermittent auscultation practices and language used by community birth midwives across the country. Current standards, as defined by midwifery schools, state midwifery licensing boards, and individual midwifery practices, differ significantly and sometimes contradict each other. Community birth midwives, nurses and birth assistants, midwifery educators and those working in community birth quality improvement have been in need of common language and guidance on best practices in intermittent auscultation.

**Objective:**

Develop and disseminate consensus standards for intermittent auscultation in the community birth setting in the United States.

**Methodology:**

Creation of guidelines through a 21‐month consensus process with a workgroup of educators, leaders, quality improvement experts, and practicing midwives by identifying practices supported by evidence or clinical experience, evaluating current evidence and guidelines, eliciting feedback from education, midwifery, nursing, and birth center organizations, and incorporating revisions to create the final document.

**Results:**

Consensus was reached on various elements of intermittent auscultation and guidelines were created. These guidelines address readiness, assessment, interpretation, and documentation of fetal heart tones, clinical decision making, and areas for future research. These guidelines provide a minimum standard for performance and documentation of intermittent auscultation in community birth midwifery practice.

## Graphical abstract

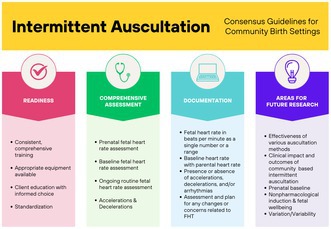

## 1 Introduction

These guidelines provide consensus recommendations for intermittent auscultation in the community birth setting in the United States. Intermittent auscultation is the gold standard for fetal assessment in uncomplicated pregnancies and labors and is used universally in the community birth setting [1, 2, 3, 4, 5, 6] including home and freestanding birth centers. A great deal of variation exists in intermittent auscultation practices and language used by community birth midwives across the country. Current standards, as defined by midwifery schools, state midwifery licensing boards, and individual midwifery practices, differ significantly and sometimes contradict each other.

Experiences with sentinel event reviews, community interaction, and teaching continuing education on fetal assessment reveal widespread confusion and quality of care concerns among community midwives related to intermittent auscultation. At the same time, published intermittent auscultation guidelines have focused on the hospital birth setting and may not be fully applicable to community birth due to available resources and unique clinical decision‐making considerations. Community birth midwives, nurses and birth assistants, midwifery educators, and those working in community birth quality improvement have been in need of common language and guidance on best practices in intermittent auscultation. This document is a response to this need. It provides a minimum standard for intermittent auscultation and documentation of auscultation in community birth midwifery practice. These guidelines should be considered alongside local rules or practice standards. They are meant to support midwifery practice and will be revised as evidence and practices evolve.

These community birth‐specific guidelines are informed by the following key documents about intermittent auscultation and the additional references cited throughout this document:

- American College of Nurse Midwives [3]. Intermittent auscultation for intrapartum fetal heart rate surveillance. ACNM Clinical Bulletin Number 60, September/October 2015. *Journal of Midwifery & Women's Health*, 60(5).
- Association of Women's Health, Obstetric and Neonatal Nurses (AWHONN) Wisner and Holschuh [1]. Fetal heart rate auscultation. *Nursing for Women's Health*, 22(6), e1–e32.

*Note on gendered language*: In recognition that women, transgender, and genderqueer people with a wide range of gender identities give birth, we use both gender neutral language (pregnant person, parental) and female gendered language (mother, maternal) in this document.

## 2 Methods

Based on an established need, a consensus workgroup was assembled. This workgroup included six midwifery leaders, educators, quality improvement experts, and practicing midwives. They were selected based on professional recommendations and credentials that applied to the goals of this project. Twenty‐three workgroup meetings were held with a quorum of four members. Existing research was evaluated, and areas for consensus development were identified. Through discussion, consensus proposals were created. Each proposal was assessed by the members on a consensus scale of 0–5, with 5 being complete agreement and 0 being a block. If a proposal was blocked or the group did not reach a consensus agreement of at least 4, it was not included in the guidelines.

Once the initial draft of the document was completed, it was sent out to a wider audience of midwifery, birth center, and nursing organizations for review. An open online forum was held for feedback and individual meetings were held with key stakeholders. Information gleaned from this process was discussed by the workgroup and revisions were completed through consensus. The final document was evaluated and approved unanimously by the workgroup.

## 3 Readiness

Three elements of readiness must be in place for effective intermittent auscultation in the community birth setting: training, equipment, and client education with informed choice.

### 3.1 Training

We recommend that:

- Midwifery schools and midwifery preceptors provide comprehensive and consistent educational and clinical training on intermittent auscultation
- Birth assistant trainings provide necessary and consistent training on intermittent auscultation
- Practicing midwives access continuing education to stay current with evolving evidence on intermittent auscultation and assessment of fetal well‐being
- Midwives share cases related to fetal assessment in peer review for mutual learning and quality improvement

### 3.2 Equipment

Community birth practices should have the following equipment available for intermittent auscultation:

- Doppler
- Doppler gel
- Extra doppler batteries in birth kit
- Watch or clock with a seconds sweep hand or digital device with seconds counter
- Fetoscope

### 3.3 Client Education With Informed Choice

We recommend that:

- During the prenatal period and in preparation for labor, midwives provide client education and informed choice about intermittent auscultation in labor [7], including:
  ○ Purpose of fetal assessment
  ○ Frequency and duration of listening in labor
  ○ Limitations of intermittent auscultation and indications for hospital transfer for continuous fetal monitoring [8]
  ○ Relevant license, certification, or practice requirements for fetal monitoring
- Consent conversations include the potential need for transfer for continuous electronic fetal monitoring if risk status changes

## 4 Assessment

Fetal heart tones are one important factor for determining fetal wellbeing. The term is used to refer to the range of information measurable through listening to the fetal heart including: baseline, presence or absence of accelerations, presence or absence of decelerations, variation in fetal heart rate, and presence or absence of arrhythmia.

### 4.1 Prenatal Fetal Heart Tones Assessment

Fetal heart rate should be assessed during prenatal care, including assessment of prenatal baseline close to term. All aspects of prenatal fetal assessment are important but are outside the scope of these guidelines.

### 4.2 Comprehensive Fetal Heart Tones Assessment

Comprehensive assessment of FHTs includes:

- Baseline fetal heart rate (FHR) assessment with parental heart rate
- Routine fetal heart tone assessment including:
  ○ Fetal heart rate
  ○ Presence or absence of accelerations [9]
  ○ Presence or absence of decelerations
  ○ Presence or absence of arrhythmia [10]

Some midwives also include variation (see Section 4.3.2) in fetal heart rate in their assessment.

### 4.3 Baseline Fetal Heart Rate

*Note*: Baseline FHR assessment is a separate component of FHT than routine FHR assessment. Please see the section below on FHT assessment in labor.

Baseline FHR [11] should be established:

- During the initial labor assessment
- At least every 4 h during active labor
- With changes such as rupture of membranes or the presence of a new risk factor

Calculation of FHR baseline [9]:

- Count FHR for a cumulative total of at least 2 min
- The 2 min of listening intervals may be consecutive or non‐consecutive as long as they are in the same 10‐min period of time
  ○ Counting in 30 or 60 s intervals may improve the accuracy of baseline FHR
- Any significant increases or decreases in fetal heart rate should not be included in calculation of baseline rate
- Fetal heart rate during fetal movement or uterine contractions should not be included in baseline
- Average the counts to determine a single number of beats per minute (e.g., 142 bpm)
- This can be accomplished with Doppler, fetoscope, or Pinard horn [11, 12]

Interpretation of FHR baseline [13]:

- Normal fetal baseline: 110–160 bpm
- Abnormal fetal baseline:
  ○ Tachycardia: 161 bpm and above longer than 10 min
    ■ May be associated with maternal fever, dehydration, infection, exhaustion, fetal infection, and/or other complications
  ○ Bradycardia: 109 bpm and below longer than 10 min
    ■ May be associated with compromised fetal status, fetal hypoxia, partial placental abruption, maternal hypotension, uterine rupture, and/or other complications

#### 4.3.1 Changes in Baseline

Some shifts in fetal heart rate baseline in labor can be normal but changes greater than 20 beats per minute can be cause for concern and warrant closer observation [14]. Significant changes in baseline can be incremental, occurring over many hours, so it is important to evaluate baseline in the full context of both the pregnancy and labor.

- Increase in baseline of more than 20 beats per minute could be an indication of maternal fever, dehydration, exhaustion, or infection and/or fetal infection and/or distress
- Decrease in baseline of more than 20 beats per minute could be an indication of fetal distress, partial placental abruption, maternal hypotension, or uterine rupture

*Accelerations* [11, 13]

- An acceleration is a temporary increase in fetal heart rate of at least 15 beats per minute lasting at least 15 s
- Accelerations are typically a sign of fetal well‐being
- Assessment should include:
  ○ Peak and duration
  ○ Frequency and pattern
  ○ Relationship to contractions and/or fetal movement

*Decelerations* [11, 13, 15, 16]

- A deceleration is a temporary decrease in fetal heart rate of at least 15 beats per minute below baseline lasting at least 15 s
- Decelerations may indicate decreased fetal blood flow, vagal response, or fetal compromise
- Assessment should include:
  ○ Nadir and duration
  ○ Frequency and pattern
  ○ Relationship to contraction (if any)
- If a deceleration is assessed:
  ○ Increase frequency and duration of IA, or listen continuously, as indicated
  ○ Change position
  ○ Make assessment of FHT pattern and determine potential causes
  ○ Rule out maternal circulatory sounds (pulse)
  ○ Assess any other risk factors
  ○ Make and implement clinical plan, including transfer if indicated

We advise against the use of vague language in the record such as “decrease” when a change in FHT meeting the definition of a deceleration is heard. While descriptions of the assessment should be included in documentation, classification of decelerations as early, late, or variable are visual assessments done with continuous electronic fetal monitoring and, as intermittent auscultation is an auditory assessment, should not be included.

#### 4.3.2 Variability and Variation

Variability, as measured visually by electronic fetal monitoring, cannot be assessed with intermittent auscultation with a doppler or fetoscope. While there is no research in this area, many community midwives find that potentially valuable information regarding fetal well‐being may be obtained by assessing for variations in fetal heart rate with intermittent auscultation [17]. Further research is needed in this area before any recommendations can be made.

In this document we use the term “variation” to refer to fluctuations in fetal heart rhythm that may be heard with intermittent auscultation.

- Practices for assessing variation in the fetal heart rate with intermittent auscultation vary
  ○ Some midwives record 2 min of 5 s counts (pausing during contractions and fetal movement) and note how frequently the 5 s counts vary above or below the baseline
  ○ Some midwives record the range of fetal heart rate heard during each episode of assessment to represent the variation or lack of variation in that episode (e.g., FHR: 136–144)
  ○ Some midwives track the variation or lack of variation in fetal heart rate over time and include this in their risk assessment
    ■ For example, if the FHR in the second stage was 128 at 7:08, 7:17, and 7:24, that may represent a different clinical picture than if the FHR was 136 at 7:08, 128 at 7:17, and 132 at 7:24.
- Research is needed to validate methods of assessing variation in the fetal heart rate with intermittent auscultation and their ability to identify fetuses at higher risk of poor outcomes.

### 4.4 Labor FHT Assessment

*Note*: Routine FHR assessment is a separate component of FHT than Baseline FHR assessment. Please see the Section 4.3 for more information.

#### 4.4.1 Initial FHT Assessment in Labor

- Assess FHT as soon as possible upon arrival
- Perform Leopold's maneuver to identify fetal position and listen as close to the fetal heart as possible to ensure assessment of heart sounds instead of cord or placental sounds
- Listen for a minimum of 2 min to establish a baseline. See Section 4.3
  ○ Do not assess the baseline rate during contractions, fetal movement, other stimuli, acceleration, or deceleration
- Note any accelerations, decelerations, or reactivity to stimuli such as contraction or fetal movement
- Check and document the birthing person's pulse to differentiate from the fetal heart rate

#### 4.4.2 Routine FHT Assessment in Labor

- At least every 30 min during active labor
  ○ Listen for at least 30 to 60 s
  ○ Listen before, during, and after the contraction at least every 60 min with the understanding that it is not always possible to listen during a contraction
- At least every 15 min during second stage
  ○ Listen for at least 30–60 s
  ○ Listen before, during, and after the contraction at least every 30 min with the understanding that it is not always possible to listen during a contraction
- Increase frequency and duration of FHT assessment if clinical or medical history indications are present
- If an attempt to auscultate FHT is unsuccessful due to the position of the birthing person, consent, noise, location, or other cause, document the reason and attempt to auscultate again when feasible
- If midwife is present with client before active labor, listen at least every 60 min
- Consider using a fetoscope to clarify the presence of an irregular fetal heart rhythm
- The digital readout on a doppler can be inaccurate so FHT assessment should rely on counting rather than the readout
- This consensus workgroup was not able to reach agreement on a more detailed recommendation of frequency and when to listen in relation to the contraction
  ○ Some of the professional organizations that have set standards for this recommend listening every 5–15 min during second stage and listening immediately after the contraction or during and after the contraction during each assessment [3, 18]

#### 4.4.3 Counting Methods

Community birth practices in the United States use a variety of counting methods to monitor fetal heart tones. There is no current evidence to support one method of counting over another.

- Some count for 5 s intervals and record those numbers serially to detect variation and multiply the numbers by 12 to calculate the fetal heart rate in beats per minute (see Section 4.3.2)
- Some count for 6 s intervals and record those numbers serially to detect variation and multiply the numbers by 10 to calculate the fetal heart rate in beats per minute
- Some count for 15 s intervals and record those numbers serially to detect variation and multiply the numbers by four to calculate the fetal heart rate in beats per minute
- Some simply include a range for the entire auscultation period (e.g., 132–144)
- There are strengths and weaknesses to each approach
  ○ Calculation of the beats per minute is simplest with 6 s counts
  ○ Shorter counts may be more effective for identifying variation in the fetal heart rate
  ○ Shorter counts may lead to more errors due to missed beats at the transition between serial intervals
  ○ Longer counts may mask variation that is present in the fetal heart rate
- Whatever method of counting is used:
  ○ A FHR in beats per minute should be recorded each time FHR is assessed
  ○ If 5‐, 6‐, or 15‐ s counts are recorded, the counting method should be identified in the chart with a key for translating the 5‐, 6‐, or 15‐ s count numbers to beats per minute.
- Example of 5‐s count key with count and corresponding rate:

**Table:** 

Count	5	6	7	8	9	10	11	12	13	14	15
Rate (bpm)	60	72	84	96	108	120	132	144	156	168	180

Difficulty with FHT assessment:

- If unable to find FHT during an auscultation attempt:
  ○ Change position and repeat attempt
  ○ Expand area on abdomen/pelvis for auscultation
  ○ Perform Leopold maneuvers to reassess fetal position
  ○ Assess other risk factors
  ○ Make and implement a plan of care, including transfer if indicated
- If an attempt to auscultate FHT is unsuccessful due to the birthing person's position, consent, noise, or location:
  ○ Discuss informed choice about fetal assessment
  ○ Document attempt and reason unable to auscultate
  ○ Repeat attempt to auscultate

#### 4.4.4 Classification Systems

The 3‐category NICHD Fetal Heart Rate classification system [13] was developed for and has only been studied in relation to continuous electronic fetal monitoring [9]. Therefore, we do not recommend the use of the NICHD categories for intermittent auscultation. Although the 3‐tier NICHD fetal heart rate categories are not directly applicable to the community birth setting, familiarity with the categories and the evidence supporting them is useful for understanding indicators of fetal well‐being [19, 20, 21].

The Institute for Perinatal Quality Improvement and the Association of Women's Health, Obstetric, and Neonatal Nurses offer a 2‐category classification system for use with intermittent auscultation [1, 22]. The intent of this system is to identify fetuses that may be at greater risk of poor outcomes and to standardize language for communication about fetal status. Research is needed to validate the 2‐category classification system [22].

## 5 Documentation

Comprehensive assessment and documentation of FHT in labor should include:

- Fetal heart rate in beats per minute as a single number or a range (e.g., 128 or 136–144)
- Baseline heart rate with parental heart rate
- Presence or absence of accelerations
- Presence or absence of decelerations
- Presence or absence of arrhythmia
- Assessment and plan for any changes or concerns related to FHT

Some midwives may also include variation in fetal heart rate in their documentation.

### 5.1 Baseline

Baseline FHR in beats per minute as a single number (e.g., 132) at least every 4 h should be included in the client record along with parental heart rate and charting should include assessment and plan when significant changes in baseline FHR are detected. Document FHR baseline as a single number (e.g., 136 bpm) not as a range (e.g., 132–140 bpm or 130 s). Include a key for translating documented 5‐, 6‐, or 15‐s count numbers to beats per minute if used.

Baseline documentation examples:

- FHR auscultated with Doppler over two consecutive minutes between contractions, using 60‐s counts; baseline FHR determined to be 142 bpm. Client position: Hands and knees. Pulse: 92 bpm.
- Fetal heart rate baseline: 142 bpm

### 5.2 Accelerations

Accelerations should be noted in the labor record including:

- Peak and duration
- Frequency and pattern
- Relationship to contractions and/or fetal movement
- Assessment and plan

### 5.3 Decelerations

Decelerations should be noted in the labor record including:

- Nadir and duration
- Frequency and pattern
- Relationship to contraction (if any)
- Assessment
- Assessment and plan

We advise against the use of vague language in the record such as “decrease” when a change in FHT meeting the definition of a deceleration is heard.

### 5.4 Difficulty With FHT Assessment

If an attempt to auscultate FHT is unsuccessful due to position, consent, noise, location:

- Document attempt and reason unable to auscultate

## 6 Clinical Decision‐Making

Intermittent auscultation is an essential tool for fetal assessment that provides community midwives with information for clinical decision‐making. An abnormal fetal heart rate, pattern, and/or rhythm are important findings that may indicate the need for clinical action in the community setting or transfer to the hospital, depending on the finding and the rest of the clinical picture. Clinical decision‐making in the presence of abnormal fetal heart tones must consider the full clinical scenario of the birthing person and the fetus, including FHT, medical history, complications of the pregnancy and labor, imminence of birth, distance from the hospital, care available at the hospital, client values and decision‐making, and more. These guidelines cannot cover the complexity of clinical decision‐making in each situation but recognize this as an important area for primary midwifery education, peer review, and continuing education.

### 6.1 When Intermittent Auscultation Is No Longer Appropriate

There are situations where intermittent auscultation is no longer appropriate and transfer to the hospital for continuous fetal monitoring is indicated. The following indications are not inclusive for continuous fetal monitoring in the hospital but are important for community midwives to recognize and respond to in conjunction with other risk factors:

- Decelerations that do not resolve with position change or other interventions available in the community setting when birth is not imminent
- Fetal tachycardia or bradycardia that does not resolve with interventions available in the community setting when birth is not imminent
- Inability to auscultate
- Inability to differentiate fetal and maternal heart rates

There are also other situations where continuous fetal monitoring in the hospital may be indicated, based on the clinical picture and the midwife's clinical judgment.

### 6.2 Labor Encouragement/Non‐Pharmacological Inductions

Many midwives use non‐pharmacological methods to encourage or induce the onset of labor, such as castor oil, uterine stimulant herbs, balloon catheter, and/or nipple stimulation in the community setting. There is limited evidence about these methods of labor encouragement, their effects on the fetus and fetal heart tones, and whether or how frequently fetal assessment is needed when they are used. We encourage community midwives to:

- Include information about the limited evidence about monitoring with labor encouragement or non‐pharmacological induction in informed choice conversations and documents
- Consider periodic intermittent auscultation or other methods of fetal assessment, depending on the complete and evolving clinical picture

### 6.3 Continuous Electronic Fetal Monitoring (CEFM)

Continuous electronic fetal monitoring in labor is inappropriate for community birth settings. Use of an electronic fetal monitor for a Non‐Stress Test or for an admission strip is not CEFM and is routine in some community birth practices [5]. Please note that the use of an EFM admission strip does not improve fetal outcomes and can result in a higher cesarean section rate [23]. When CEFM is indicated, the hospital is the appropriate setting for labor and birth.

## 7 Areas for Future Research

More research is needed on intermittent auscultation and specifically on intermittent auscultation in the community setting. Research is especially needed in the following areas:

- Methods of FHT assessment—Research is needed on the most effective frequency, timing, counting method, and duration of FHT assessment with intermittent auscultation in each birth setting
- Intermittent auscultation and outcome—Research is needed on the clinical impact and associated outcomes with intermittent auscultation in community birth settings
- Prenatal baseline**—**Research is needed to study the value of prenatal FHR baseline in assessing fetal wellbeing in labor
- Non‐pharmacological labor induction methods and fetal wellbeing—Research is needed about whether there are fetal effects of non‐pharmacological labor induction methods
- Variation—Validation studies are needed to evaluate methods of assessing variation in the fetal heart rate with intermittent auscultation and their ability to identify fetuses at higher risk of poor outcomes

## Conflicts of Interest

The authors declare no conflicts of interest.

## Data Availability

The authors have nothing to report.

## Appendix A

### Definitions [6, 24]

*Acceleration*: A temporary increase in FHR of at least 15 beats per minute above baseline lasting at least 15 s but no longer than 2 min. A prolonged acceleration is longer than 2 min but no more than 10 min long.

*Baseline FHR*: Mean fetal heart rate during at least 2 min of fetal heart tone assessment within a 10‐min period of time excluding time when the fetus is experiencing or reacting to stimuli such as contraction, fetal movement, or external palpation.

*Birth assistant* [25, 26, 27]: An individual who is not a trained midwife but who assists a midwife at births for compensation.

*bpm*: beats per minute.

*Bradycardia*: Baseline fetal heart rate of less than 110 beats per minute for 10 min or more.

*Continuous electronic fetal monitoring* (CEFM): A method of measuring and recording the fetal heart rate and the frequency of uterine contractions continuously using external or internal sensors.

*Deceleration*: A temporary decrease in FHR of at least 15 beats per minute below baseline lasting at least 15 s but no longer than 2 min. A prolonged deceleration is longer than 2 min but no more than 10 min long [9, 28, 29, 30].

*Fetal heart rate* (FHR): The rate of fetal heartbeats is measured in beats per minute.

*Fetal heart tones* (FHT): Term used to refer to the range of information measurable through listening to the fetal heart including: baseline, presence or absence of accelerations, presence or absence of decelerations, variation in fetal heart rate, and presence or absence of arrhythmia.

*Nadir*: The nadir is the lowest point, or slowest fetal heart rate recorded during a period of FHR assessment. Most often used to refer to the lowest point in a deceleration.

*Reactivity*: Fetal heart rate acceleration response to movement, contraction or other stimulation which is an indicator of fetal well‐being.

*Tachycardia*: Baseline fetal heart rate of more than 160 beats per minute for 10 min or more.

*Variability*: Fluctuations in baseline fetal heart rate that can be visually quantitated [3].

*Variation*: Fluctuations in fetal heart rate that may be heard with intermittent auscultation as opposed to visually quantitated.

## Appendix B

### Additional Resources

PQI intermittent auscultation simulation‐based education: https://www.perinatalqi.org/page/iaedfaqs.

Hive continuing education fetal assessment in labor course with Holly Scholles: https://www.hivece.com/courses/fetal‐assessment‐in‐labor‐2.

2018 NACPM Webinar: Intermittent auscultation with Wendy Gordon: https://www.nacpm.org/intermittent‐auscultation.

Evidence based birth. The evidence on fetal monitoring: https://evidencebasedbirth.com/fetal‐monitoring/.

ACNM clinical bulletin. Intermittent auscultation for intrapartum fetal heart rate surveillance: https://www.cmqcc.org/resource/3223/download.

ICM position statement. Use of intermittent auscultation for assessment of foetal wellbeing during labour: https://www.internationalmidwives.org/assets/files/statement‐files/2018/04/eng‐use_intermittend_auscultation.pdf.
